# Modeling and measurement of the variations of CT number distributions for mobile targets in cone‐beam computed tomographic imaging

**DOI:** 10.1120/jacmp.v16i1.5067

**Published:** 2015-01-08

**Authors:** Imad Ali, Nesreen Alsbou, Salahuddin Ahmad

**Affiliations:** ^1^ Department of Radiation Oncology University of Oklahoma Health Sciences Center Oklahoma City OK; ^2^ Department of Electrical and Computer Engineering Ohio Northern University Ada OH USA

**Keywords:** cone‐beam CT, CT number distribution, respiratory motion, motion model, image artifacts

## Abstract

The purpose of this study was to investigate quantitatively by measurement and modeling the variations in CT number distributions of mobile targets in cone‐beam CT (CBCT) imaging. CBCT images were acquired for three targets manufactured from homogenous water‐equivalent gel that was inserted into a commercial mobile thorax phantom. The phantom moved with a controlled cyclic motion in one‐dimension along the superior–inferior direction to simulate patient respiratory motion. Profiles of the CT number distributions of the static and mobile targets were obtained using CBCT images. A mathematical model was developed that predicted the variations in CT number distributions and their dependence on the motion parameters of targets moving in one‐dimension using CBCT imaging. The measured CT number distributions for the mobile targets varied considerably, depending on the motion parameters. The extension of the CT number distribution increased linearly with motion amplitude where maximum target elongation reached twice the motion amplitude. The CT number levels of the mobile targets were smeared over a longer distribution; for example, the CT number level for the 20 mm target dropped by nearly 30% at motion amplitude (A) equal to 20 mm in comparison with the CT number distribution of stationary targets. Frequency of motion played an important role in spatial and level variations of the CT number distributions. For example, the level of the CT number profile for the medium target (20 mm) decreased evenly by nearly 50% at A=20 mm with high motion frequencies. Motion phase did not affect the CT number distributions for prolonged projection acquisition that included several respiratory cycles. The mathematical model of the CT number distributions of mobile targets in CBCT reproduced well the measured CT number distributions and predicted their dependence on the target size and phantom motion parameters such as speed, amplitude, frequency, and phase. The CT number distributions varied considerably with motion in CBCT. A motion model of CT number distribution for mobile targets has been developed in this work that predicted well the variations in the measured CT number profiles and their dependence on motion parameters. The model corrected the CT number distribution retrospective to CT image reconstruction where it used a first‐order linear relationship between the number of projections collected in the imaging window of a mobile voxel to obtain the cumulative CT number. This model provides quantitative characterization of motion artifacts on CT number distributions in CBCT that is useful to determine the validity of CT numbers and the accuracy of localization and volume measurement of tumors in diagnostic imaging and interventional applications, such as radiotherapy.

PACS number: 87.57.C‐

## I. INTRODUCTION

CT imaging plays an essential role in the diagnosis of various diseases, including cancer, where it provides a valuable tool in screening and staging of various cancers.^1^, ^2^, ^3^ In radiotherapy, CT imaging has a paramount role where the tumor and critical structures are usually outlined in the treatment planning process.^4^, ^5^ However, patient motion degrades the quality of CT images.^6^, ^7^, ^8^ Different techniques were introduced to handle motion artifacts in CT imaging. These techniques include rapid gantry rotations combined with multislice technology to achieve shorter scanning with less motion artifacts^9^, ^10^ and correction of motion artifacts in the projections^11^, ^12^ before image reconstruction, where the trajectory of a mobile object is remapped back to the stationary positions. 4D CT is used to account for patient motion^13^, ^14^, ^15^, ^16^ in which the projections are sorted into different respiratory motion phases and CT images are reconstructed in each phase with reduced motion artifacts.

Cone‐beam computed tomographic imaging (CBCT) provides a robust tool for volumetric tomography using high resolution and sensitive flat‐panel detectors.^17^, ^18^ An X‐ray source provides three‐dimensional cone beams that are detected with a large effective imaging area using flat‐panel detectors^(18)^ or multiple detector arrays.^19^, ^20^ In CBCT, the projections are acquired by rotating the imaging gantry around the patient to obtain different angular views to reconstruct volumetric images of the patient.^(21)^ Over the last decade, interventional applications of CBCT have grown, where increasing number of radiation therapy machines are being equipped with kV on‐board imaging (OBI) systems that can provide planar radiographic imaging, volumetric CT imaging, and fluoroscopy.^(22)^ The OBI has become a vital clinical tool to perform image‐guided radiation therapy (IGRT). However, patient motion degrades image quality and thus limits the visibility of tumors and critical structures.^(23)^ In this work, variations in the distributions of the CT numbers for well‐defined targets induced by motion were assessed quantitatively by measurement and modeling. The changes in the extension and level of CT numbers for three mobile targets inserted in a thorax phantom were measured quantitatively by CBCT imaging using a kV on‐board imager. Controlled cyclic motion patterns were induced to simulate image artifacts from patient respiratory motion. The measured distributions of the CT numbers of the stationary and mobile targets were employed to develop a mathematical model that predicts quantitatively the motion effects on the CBCT number distributions.

## II. MATERIALS AND METHODS

### A. Phantom setup

**Figure 1:**
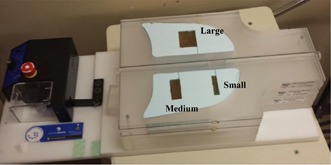
Thorax mobile phantom with water‐equivalent targets — small ($3\times 1\times 5\;{\text{cm}}^{3}$), medium ($3\times 2\times 5\;{\text{cm}}^{3}$) and large ($3\times 4\times 5\;{\text{cm}}^{3}$) — fabricated from a gel material and inserted in low‐density foam.

A thorax phantom system (Standard Imaging, Inc., Middleton, WI) was assembled with homogenous water‐equivalent targets that were inserted in the middle of lung tissue‐ equivalent medium. The phantom was then mounted on a sinusoidally moving platform, as shown in Fig. 1. The system moved with a cyclic motion in one dimension along the superior–inferior direction (y‐axis) using various motion amplitudes ranging from 0–20 mm at a frequency of 15 cycles/min during CT scanning. Three targets, small ($3\times 1\times 5\;{\text{cm}}^{3}$), medium ($3\times 2\times 5\;{\text{cm}}^{3}$), and large ($3\times 4\times 5\;{\text{cm}}^{3}$), were fabricated from a gel material and inserted in low‐density foam to simulate mobile lung tumors.

The phantom was then imaged using an on‐board imager (OBI) with kV CBCT integrated with a TrueBeam linear accelerator (Varian Medical Systems, Inc., Palo Alto, CA). The OBI consists of a kV X‐ray source with a diagnostic image quality and a flat‐panel imager (PaxScan 4030CB; Varian Medical Systems). The imager was operated in 2×2 binning mode, where the projections were acquired with a 1024×768 pixels that covered $40\times 30\;{\text{cm}}^{2}$ effective area with a spatial resolution of nearly $0.39\times 0.39\;{\text{mm}}^{2}$ at isocenter, which is located at a distance of 150 cm from the imaging X‐ray source. The OBI was employed to obtain full‐fan and half‐fan CBCT of the mobile phantom system. In full‐fan scans, a small imaging volume of 25 cm diameter and 15 cm thickness are obtained, where projections are acquired over 180° angular range. In full‐fan scans, the imager position is offset to cover a large imaging volume of 50 cm diameter and 17 cm thickness, where projections are acquired over 360° angular range. The imaging parameters used were 2 mm slices thickness, 125 kVp, and 264 mAs for half‐fan CBCT, and 2 mm slice thickness, 100 kVp, and 146 mAs for full‐fan CBCT. The phantom was scanned while it was static and moving, using the previously mentioned cyclic motion patterns. The CT images were subsequently processed with the Eclipse treatment planning system (Varian Medical Systems). Coronal image views for all scans with the different motion amplitudes from each of the two imaging modes were reconstructed and used to measure the CT number distributions and levels. To maintain consistency in the measurement of CBCT number distributions, the coronal slice that passed through the centers of all targets was selected, where all targets were set at the same level in the anterior–posterior direction, as shown in Fig. 1.

### B. Modeling of target broadening in CBCT

**Figure 2:**
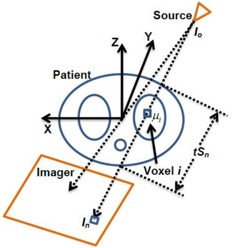
Reference coordinate system with a ray ($I_{n}$) that passes through voxel i in a mobile target.

Figure 2 shows a top view in the (X, Y, Z) coordinates with the three targets embedded in a phantom moving along the Y direction which was used in the modeling of the CT number distributions. To simplify the modeling, a stationary target composed of homogenous water‐equivalent material that sits in the middle of air is considered. If N projections are acquired during CBCT scanning of a stationary object, they are used by the CT image reconstruction algorithm to calculate CT numbers. In CBCT, a projection measures the attenuation of radiation of an object in its path, as shown in Fig. 2, where the intensity ($I_{n}$) measured at pixel *n* on the imager represents the attenuation of all voxels along a ray of radiation from the source, as follows:

(1) $$\text{ln}(\frac{I_{n}}{I_{o}})=-\sum_{i=1}^{S_{n}}\mu_{i}t_{i}$$

where $I_{o}$ is the initial intensity produced by the imaging source, $\mu_{1}$ is the linear attenuation coefficient of voxel *i* with thickness $t_{i}$, and $S_{n}$ is the number of voxels in the ray track. All voxels were assumed to have equal thickness, t, and thus ray, n, passes through a total thickness equal to ${\text{tS}}_{n}$, as shown in Fig. 2. An image reconstruction algorithm solves numerically many equations like Eq. (1) that include all pixels on the two‐dimensional images and different angular views in order to obtain the linear attenuation coefficient $\mu_{1}$ at voxel i. For example, in the filtered back‐projection image reconstruction,^(21)^ a large number of projections are back‐projected on the image matrix to build up the CT number value. Each projection contributes incrementally $\Delta CT_{n}^{i}$ to the cumulative CT number value of voxel i considering that the linear attenuation factor of this voxel is the same in all projections. This is an acceptable approximation considering negligible variations in the value of the linear attenuation coefficient for voxel i due to variations in the imaging beam energy. N is the number of projections that are acquired within an imaging window (w) for the stationary phantom; w is defined here as the spatial interval where the projections of voxel i are acquired which is assumed to be equal for all voxels. Thus, in a stationary voxel, the CT number of the stationary voxel, $CT_{s}^{i}$, is linearly proportional to the number of projections:

(2) $$CT_{S}^{i}=\Delta CT_{n}^{i}\cdot N$$

If the previous target is now moving, then the number of projections (M) acquired within the imaging window (w) for voxel i that will be used by the image reconstruction algorithm is different from the stationary target because the mobile target is moving in or out of w. The CT number ($CT_{M}^{i}$) of voxel i in the mobile target is again linearly proportional to the number of projections (M) that are acquired:

(3) $$CT_{M}^{i}=\Delta CT_{n}^{i}\cdot M$$

From the previous equations, the relationship between the CT number levels of voxel i for the mobile and stationary targets is given by:

(4) $$CT_{M}^{i}=CT_{S}^{i}\frac{M}{N}$$

**Figure 3:**
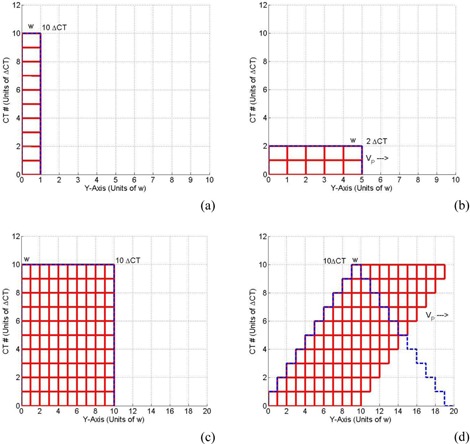
Simulation of the CT number distributions with acquisition and sorting of the projections (N=10) for one voxel target when it is stationary (a) and moving (b) with a constant speed ($V_{P}$). Simulation of the CT number distributions with acquisition of the projections (N=10) for a target made from ten voxels when it is stationary (c) and moving (d) with a constant positive speed ($V_{P}$) in the superior‐inferior direction. The cumulative CT number profiles are represented by the dashed curves.

Figure 3 represents a simulation of the variations in the CT number distributions for stationary and mobile targets using CBCT imaging. The CT numbers of voxel i=1 as a function of position for a stationary and mobile targets is represented in Figs. 3(a) and (b). In the case of stationary target, for example, all projections (N=10) are acquired by the gantry as it rotates around the target during scanning within an imaging window (w) of voxel i, as shown in Fig. 3(a), where w represents the width of one slice thickness in the CBCT images. For the mobile target, smaller number of projections (M=2) are acquired in each window and the CT number level is obtained from increments *of* $\Delta CT_{n}^{i}$ that broaden over a range of distance (5w) which depends on the speed of the mobile target, as shown in Fig. 3(b). In Fig. 3(c), the projections (N=10) of a target with ten slices (10w) is acquired for the stationary target. As the target moves, the CT numbers broaden over a longer range of slices (19w). The CT numbers of the different slices vary where the number of projections and the corresponding CT number level, as represented by the dashed curve increases, until it reaches a maximum at the tenth slice (N=10). Afterwards, it decreases, reaching zero at 20th slice.

Notice that the cumulative value of all the projections and the corresponding CT numbers from all imaging windows for one voxel (Fig. 3(a)) or for a whole target with many voxels (Fig. 3(c)) is the same for a target when it is either stationary or mobile (Fig. 3(b) and 3(d)). The areas under the stationary or mobile CT number profiles for each target are equal:

(5) $$CT_{S}^{i}w=CT_{M}^{i}(w+L_{i})$$

where $L_{i}$ is the extension of CT number in the mobile target. The CT number of voxel i for the mobile target is then given by the following:

(6) $$CT_{M}^{i}=CT_{S}^{i}\frac{w}{w+L_{i}}=CT_{S}^{i}\frac{w}{w+V_{P}T_{i}}$$

where $V_{P}$ is the speed of the mobile target and $T_{i}$ is the time needed to acquire all cone‐beam projections over a distance $L_{i}$ that the target will move to during imaging.

In the case of a stationary target, the CT number of voxel i is calculated from N projections acquired in w located at $y_{i}^{o}$ along the superior‐inferior direction during scanning. However, for a mobile target, the CT number at $y_{i}^{o}$ is obtained from the contributions of the projections of several voxels that are acquired in w as they pass during CBCT imaging. Equation (6) can be generalized to obtain the CT number of voxel i at a specific position $y_{i}$ during imaging from the contributions of different voxels of the mobile target, as given by the following:

(7) $$CT_{M}(y_{i}^{o})=\sum_{m}^{V}\sum_{n}^{N}\Delta CT(m,n)\frac{w}{w+y_{i}(t)-y_{i}^{o}}$$

where *V* is the number of voxels that belong to the target, *N* is the number of projections acquired in CBCT scanning, $y_{i}^{o}$ is the position of voxel i in stationary phantom, $y_{i}(t)=V_{P}\;t$ is the position of voxel i at time *t* which changes, depending on the speed of the mobile target ($V_{P}$ during imaging). In Eq. (7), all the voxels in the mobile target were assumed to move with a constant speed ($V_{P}$).

### C. CBCT number distribution simulation

The modeling of CT number distributions was tested using programming with MATLAB (MathWorks, Inc., Natick, MA). The composition of the three targets was considered to be water‐equivalent tissues and the CT number level was setup to be equal to the CT number level of each stationary target. The surrounding medium was considered lung tissue‐equivalent. The measured CT numbers for the same target varied by ±20 HU between half‐fan and full‐fan CBCT. The position and length of each target and motion parameters, such as motion amplitude, frequency, and phase, were input in the simulation to calculate the CT number distributions. Furthermore, imaging parameters such as slice thickness, gantry speed, imaging view, and detector resolution were input in the MATLAB program to simulate CT numbers. The calculated CT number profiles of the stationary and mobile targets with various motion patterns from simulation were compared with the measured profiles of CT numbers obtained from CBCT imaging.

## III. RESULTS

**Figure 4:**
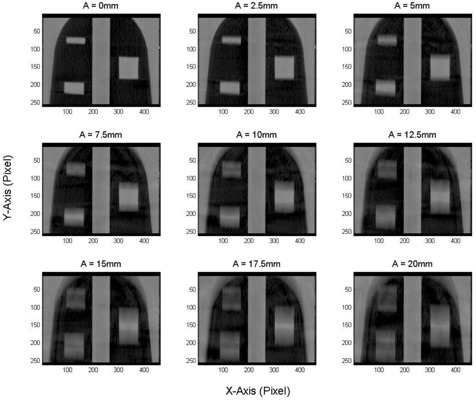
Coronal views of the CT number distributions for the three targets (small, medium, and large) with different motion amplitudes using half‐fan CBCT images. X‐ and y‐axis are represented in pixels. The targets moved cyclically in the superior–inferior direction (y‐axis) with nine different motion amplitudes: 0, 2.5, 5, 7.5, 10, 12.5, 15, 17.5, and 20 mm with a frequency of 15 cycles per min.

**Figure 5:**
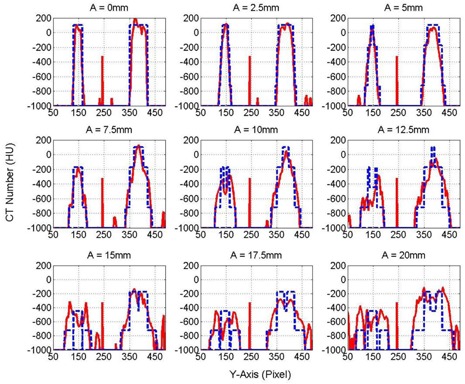
CT number profiles of the small and medium targets using half‐fan CBCT imaging with different motion amplitudes, as indicated. The solid and dashed curves represent the measured and model CT number levels, respectively.

**Figure 6:**
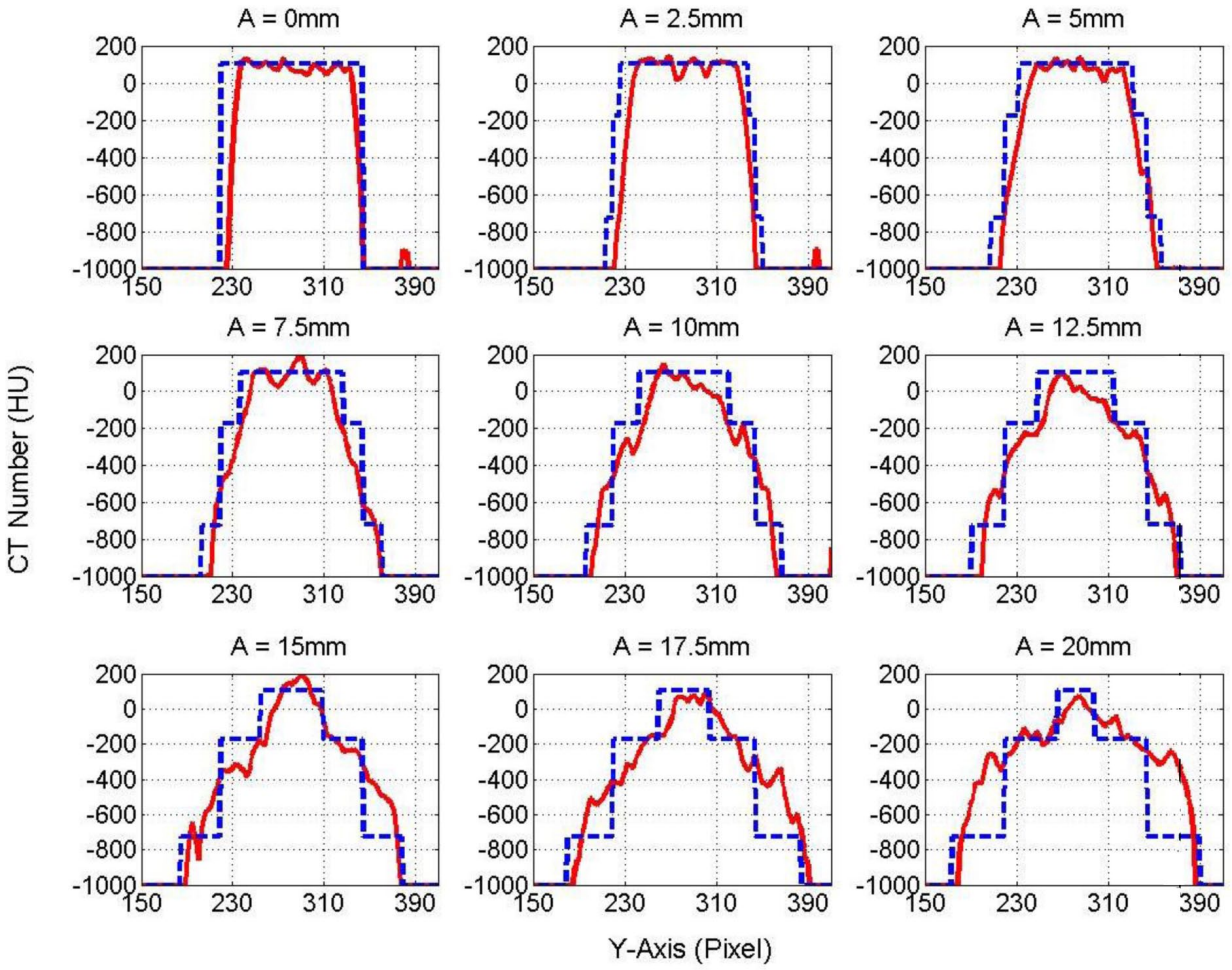
CT number profiles for the large target from half‐fan CBCT imaging with different motion amplitudes, as indicated. The solid and dashed curves represent the measured and model CT number levels, respectively.

Figure 4 shows coronal views obtained from half‐fan CBCT images of the three targets, small, medium, and large, inserted in the phantom with different cyclic motion amplitudes ranging from 0‐20 mm. The CT number distributions extended over large distances that increased linearly with the motion amplitude (A). The distributions were flat for the stationary targets made from homogenous material in this experiment. However, as the A increased, the CT number levels became less flat with maximum moving from the center to the edges of the distributions. Figure 5 shows the profiles of the CT numbers along the direction of motion (y‐axis) for the small (10 mm) and medium (20 mm) targets with different motion amplitudes, as indicated. The solid and dashed curves represent the measured CT number obtained from CBCT images and calculated profiles using Eq. (7), respectively. In the CT number distribution for the small target, the maximum of the CT number level shifted from the center of the target to the edges as the range of motion (ROM), which is twice the motion amplitude, increased from 0–40 mm. When the ROM became larger than the actual length of the small target (>10 mm), the CT number distributions split in the middle where the CT number maxima were located close to the periphery of the distributions. The maximum of the CT numbers was located centrally for the medium target with ROM <10 mm. When the ROM was larger than the length of the target (20 mm), then the CT number distribution split, with the maximum shifted towards the edges of the broadened target. Similar behavior was seen with the large target (40 mm), as shown in Fig. 6, where the maximum level of the CT numbers decreased as the distribution broadened over a larger spatial extension. 5, 6 show the variations of the CT number profiles along the direction of motion with different ROMs for the three targets. For example, the CT number level for the 20 mm target dropped by nearly 30% at ROM=40 mm, in comparison with the CT number distribution of stationary targets. Although the CT numbers broadened over larger volume and the CT number level decreased with the increase in the extension of the CT number distributions, the total cumulative CT number over the broadening range was preserved, as predicted by Eq. (5) as long as the target is in the imaging view during CBCT scanning. In Fig. 5 there were few spikes, which might be induced by streaking artifacts form dense objects or metal components in the treatment couch or phantom setup. The treatment couch is composed from a dense carbon‐fiber frame and some metal component to support patients. The thorax phantom has a metal arm and motor components that drive the mobile platform, which might produce the streaking artifacts in CBCT images as it extends in the imaging view during phantom motion.

Table 1 shows the percentage normalized root mean square (PRMS) for the difference of the calculated and measured CT number profiles for the small and medium targets presented in Fig. 5 and large target in Fig. 6. The right column represented the motion amplitude (A) of the mobile targets. The highest PRMS was 6.2% for the small target at A=12.5 mm, which is larger than the actual length of the target (10.0 mm) and which resulted from high gradient in the CT number profiles that were generated by splitting the CT number profile due to spread‐out by motion. PRMS for the small target was generally larger than the medium and large targets because of the high gradient in the CT number profiles for the small target. For the medium target, PRMS decreased as A increased until it reached intermediate values before CT number profiles split and produced high‐gradient CT number profiles when the range of motion (2A) became larger than the actual length of the medium target. As A increased, the CT number profiles for the large target became flatter without splitting the CT number profiles and, thus, PRMS decreased.

**Figure 7:**
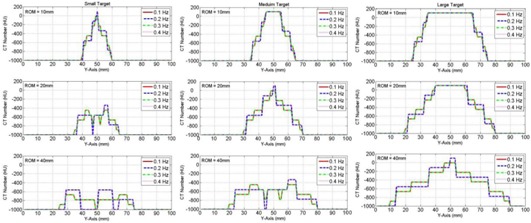
Simulation of the CT numbers profiles dependence on motion frequency for the three targets — small (10 mm) in the first column, medium (20 mm) in the second column, and large (40 mm) in the third column — moving with the indicated ranges of motion (ROM=2A).

Figure 7 shows simulation of the variations of the CT number profiles with different frequencies (0.1–0.4 Hz) and ROMs (10–40 mm) for the three targets: small (10 mm), medium (20 mm), and large (40 mm). The centers of the three targets were assumed to be at 50 mm in their stationary position, and then they were allowed to move with cyclic motion in the superior–inferior direction. At small ROMs of the mobile targets, the maximal CT number level in the profiles remained nearly the same with small variations due to changes in the motion frequency. However, at large ROM (40 mm), the profiles changed significantly, where the CT numbers were then distributed evenly over the extended ROM. For example, the level of the CT number profile for the medium target (20 mm) dropped evenly by nearly 50% at a ROM=40 mm. This is because the mobile targets were present more often in the middle of the distribution for high frequencies in comparison with that at the periphery for low frequencies where they stayed longer times turning around with low speeds. The phase of motion did not affect the CT number distributions when the projections were acquired over several motion cycles.

**Table 1:** Percentage normalized root mean square for the difference of the measured and calculated CT number profiles (small, medium, and large targets) for the motion amplitudes in the first column

*A (mm)*	*Small Target*	*Medium Target*	*Large Target*
0.0	5.4	4.2	3.0
2.5	3.2	2.7	2.4
5	3.6	2.6	1.7
7.5	2.8	2.5	1.6
10	4.7	2.6	1.7
12.5	6.2	2.8	1.7
15	5.1	2.5	1.8
17.5	5.4	2.8	1.9
20	5.3	3.7	2.2

**Figure 8:**
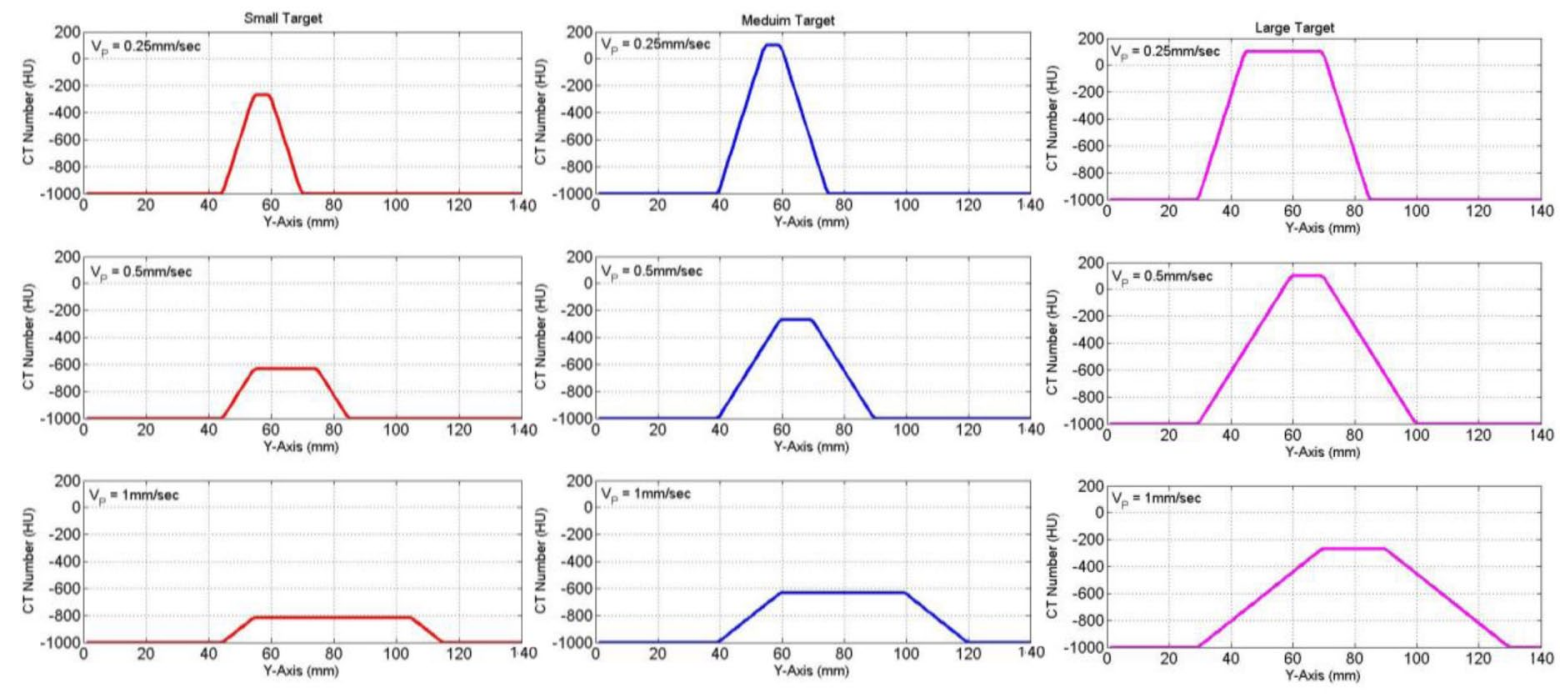
Simulation of the CT numbers distributions with unidirectional constant speeds along the superior–inferior directions: 0.25, 0.5, and 1.0 mm/sec for the three targets — small (10 mm) in the first column, medium (20 mm) in the second column, and large (40 mm) in the third column.

**Figure 9:**
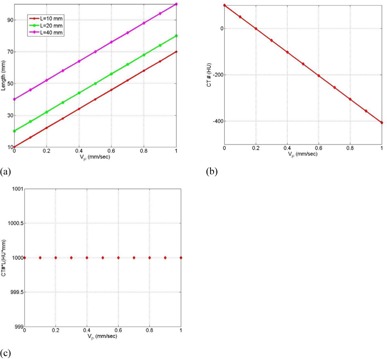
Length of the CT numbers distribution (a) for the three targets as a function of the speed of the mobile targets in CBCT imaging; decrease of the CT number level (b) at the edges as a function of the speed of the mobile target; the integral of CT numbers level (c) over the broadening distance of the mobile targets as a function of speed.

Figure 8 demonstrates the dependence of the CT number distributions of the three targets on speed where unidirectional constant speeds along the y‐axis (superior–inferior) were used. As the speed of a mobile target ($V_{P}$) increased, the CT number distribution broadened forward along the direction of motion. The elongation of the CT number distributions increased linearly with the speed of the mobile targets, as shown in Fig. 9(a). Furthermore, the CT number levels at the edge of the distributions decreased linearly with the speed of the mobile targets along the Y direction (Fig. 9b)). The CT number levels for each target decreased as the speed increased, as predicted by Eq. (5) and shown in Fig. 9(c).

## IV. DISCUSSION

In this study, a mathematical model was introduced to predict the CT number distributions of mobile targets in CBCT imaging. This model is based on considering a first‐order linear relationship between the number of projections captured in the imaging widow of a mobile voxel and the cumulative CT number obtained from image reconstruction algorithm. It was used to predict the variations of CT number distributions induced by controlled motion patterns retrospective to the image reconstruction process. The model was verified by measurement of the variations in CT number distributions of mobile targets with different controlled motion patterns and imaged using half‐fan and full‐fan CBCT imaging. The variations in the broadening and level of CT number distributions and their dependence on motion parameters were reproduced by the mathematical modeling. One limitation of this model is that the mobile targets have to stay in the cone‐beam imaging view when using the half‐ and full‐fan imaging modes. If a target moves out of the imaging view in certain projections, then the CT number level obtained from CBCT reconstruction is different. Thus the preservation of cumulative CT numbers represented in Eq. (5) is not valid. In half‐fan scanning mode, one side of the phantom is imaged and the shadow of a mobile target has to be acquired in the projections needed to reconstruct CBCT images for that specific side. For the same motion patterns, the CT number distributions in full‐fan CBCT are similar to those seen in half‐fan CBCT as long as the mobile target does not shift out of the half‐fan beam imaging view. Furthermore, the measured CT number distributions were limited by detectors size, electronic noise, volume averaging, and approximations used in the CBCT image reconstructions;^(21)^ however, this motion model did not consider these effects. Although, the modeling in this study is limited to one‐dimensional motion along the superior–inferior directions and stationary imaging couch, it can be generalized to other realistic complicated motion patterns that may be the subject of a future study.

CBCT is routinely used as interventional tool in IGRT;^(22)^ however, elongation of a tumor by patient motion affects the localization accuracy and patient setup. In this study, controlled cyclic motion was employed with known motion amplitude and frequency. In a real patient, these motion parameters are unknown. The modeling developed here has potential clinical application in IGRT where it provides a method to extract unknown motion parameters from the measured CT number distributions and levels. For example, if a patient has an implanted marker or another anatomical surrogate with known initial length and composition, then the broadening or elongation of the nearby tumor can be determined, assuming that the tumor broaden in the same way as the marker. According to Eq. (5), the elongation of the tumor can be used to determine motion amplitude. Motion frequency may be extracted from the CBCT number distributions; however, this needs further investigation.

The use of CBCT images is limited by image artifacts induced by scatter radiation,^(24)^ approximations of image reconstruction algorithm,^(21)^ and patient motion.^8^, ^23^, ^25^, ^26^ Thus, CBCT is not used currently as standard in contouring and dose calculation in treatment planning systems.^(27)^ Of particular interest is scattered radiation, which is a random process that enhances the noise and lower contrast in CBCT imaging without producing patterns of image artifacts. Thus, scattering artifacts might be distinguished from the ones associated with motion that produce certain patterns such as spreading out the CT number distribution, blurring at the edges of a sharp object, and a dropping in the CT number level. The uncertainty in CBCT number could be as large as ± 40 HU, based on the extreme vendor acceptance criteria; however, in our CBCT imaging system, uncertainly is often within ±20 HU. New CT scanners use multiple array detectors to image large portions of the patient that combine CBCT and helical or axial CT techniques. In these diagnostic CBCT imaging systems, the variations in the CT number distribution and tumor density due to patient motion influence the accuracy of disease diagnosis. The employment of CBCT in radiotherapy will affect the accuracy of the treatment planning, dose calculation and patient setup. The variations in CBCT number distribution and level by patient motion may cause wrong portrayal of tumor volume. The increase in the extension of the CBCT number distributions of the tumor volume will cause outlining of less tumor or inclusion of normal tissue on the CT images used by the treatment planning system.^4^, ^5^, ^28^ Furthermore, variations in the CT number density cause changes in the electron density used to correct heterogeneity by the dose calculation algorithm.^29^, ^30^

## V. CONCLUSIONS

A mathematical model has been developed in this study that predicted the CT number distributions of mobile targets in CBCT imaging. The model considered first‐order linear relationship between the number of projections collected in the imaging widow of a mobile voxel and the cumulative CT number obtained from image reconstruction algorithm. The model predicted well the variations of CT number distributions induced by controlled motion patterns retrospective to the image reconstruction process. The variations in CT number distribution need to be considered in diagnostic CT imaging in order to achieve accurate tumor localization and volume measurement. The motion‐induced artifacts in the CT number distributions have to be accounted for in radiation therapy in order to outline the actual tumor volumes, use accurate CT numbers in treatment planning, and set up patients and localize tumors using IGRT based on CBCT.
